# St. Bartholomew's Hospital Appeal

**Published:** 1909-10-30

**Authors:** 


### ST. BARTHOLOMEW'S HOSPITAL APPEAL.

A few months ago public attention was directed to the needs of St. Bartholomew's Hospital by a conference held at the Mansion House between the Lord Mayor, the Hospital Treasurer, and the masters of leading City Guilds, with reference to the financial position of the institution. There is now an annual deficit of £12,000, due largely to the payment of interest on a capital debt of £170,000. At the conference it was decided to wait till the autumn before issuing an appeal to the public for support. That appeal, numerously signed, has now been made; and a special fund started to liquidate the capital debt and thus reduce the annual deficit. Cheques may be addressed to the Lord Mayor, Mansion House, E.C., or to Lord Sandhurst, Treasurer, St. Bartholomew's Hospital, E.C. The fact that the hospital has been maintained for generations unaided by the public, and that it has not participated in the benefactions of King Edward's Hospital Fund for London or the Hospital Sunday and Saturday Funds, should make this appeal of interest to every citizen. Lord Sandhurst has thrown himself enthusiastically into the matter, and his efforts on behalf of our oldest Metropolitan institution have already borne fruit in the sums promised or subscribed to the appeal.

